# Circulating Interleukins-33 and -37 and Their Associations with Metabolic Syndrome in Arab Adults

**DOI:** 10.3390/ijms25020699

**Published:** 2024-01-05

**Authors:** Osama E. Amer, Shaun Sabico, Malak N. K. Khattak, Abdullah M. Alnaami, Gamal M. Saadawy, Nasser M. Al-Daghri

**Affiliations:** Chair for Biomarkers of Chronic Diseases, Biochemistry Department, College of Science, King Saud University, Riyadh 11451, Saudi Arabia

**Keywords:** diabetes, cardiovascular diseases, inflammation, Arab, metabolic syndrome

## Abstract

Interleukins (ILs) are a group of cytokines known to have immunomodulatory effects; they include ILs–33 and –37 whose emerging roles in the pathogenesis of metabolic syndrome (MetS) remain under investigated. In this study, we compared circulating IL–33 and IL–37 in Arab adults with and without MetS to determine its associations with MetS components. A total of 417 Saudi participants (151 males, 266 females; mean age ± SD 41.3 ± 9.0 years; mean body mass index ± SD 30.7 ± 6.3 kg/m^2^) were enrolled and screened for MetS using the ATP III criteria. Anthropometrics and fasting blood samples were taken for the assessment of fasting glucose and lipids. Circulating levels of IL–33 and IL–37 were measured using commercially available assays. The results showed higher levels of serum IL–33 and IL–37 in participants with MetS than those without (IL-33, 3.34 3.42 (2.3–3.9) vs. (1–3.9), *p* = 0.057; IL-37, 5.1 (2.2–8.3) vs. 2.9 (2.1–6.1), *p* = 0.01). Additionally, having elevated levels of IL–33 was a risk factor for hypertension, low HDL-c, and hypertriglyceridemia. A stratification of the participants according to sex showed that males had higher IL-33 levels than females [3.7 (3.0–4.1) vs. 3.15 (1.4–3.8), *p* < 0.001], while females had higher levels of IL–37 than males [3.01 (2.2–7.0) vs. 2.9 (2.1–5.6), *p* = 0.06]. In conclusion, the presence of MetS substantially alters the expression of ILs–33 and -37. IL-33 in particular can be potentially used as a therapeutic target to prevent MetS progression. Longitudinal and interventional studies are warranted to confirm present findings.

## 1. Introduction

MetS is a group of cardio metabolic risk factors that include dyslipidemia, central obesity, insulin resistance, and hypertension. Individuals with MetS are at a greater risk of developing T2DM and CVD [1]. MetS is a state of chronic low-grade inflammation evidenced by increased levels of numerous inflammatory biomarkers, e.g., tumor necrosis factor α (TNF-α), interleukin 1β, and C-reactive protein [2,3]. Previous studies have demonstrated that adipose depots, the intestine, and the liver are the main sites that trigger MetS-associated inflammation [4,5,6]. MetS is considered to be a response to metabolic stress resulting from chronic caloric excess and subsequent cell death [7,8,9]. Inflammatory factors released from one site can cascade into other sites, subsequently increasing the chronic inflammatory state and generalized tissue dysfunction/damage [6]. Understanding the modulation of this inflammatory state could assist in ameliorating the detrimental effects of MetS and its associated consequences. In Saudi Arabia, obesity-mediated MetS is a public health burden with extensive economic impact [10,11]. The prevalence of MetS in Saudi Arabian adults was 39.8% in 2018 [12].

Interleukin-33 (IL–33) is a recently identified alarmin cytokine from the IL-1 family. It has a vital role in the induction of immune responses as well as in metabolism regulation [13]. Zeyda et al. demonstrated increases in IL–33 mRNA and protein expression in the omental adipose tissue of obese subjects as compared to lean/overweight individuals [14], which was also observed in mice [15]. IL–33 expression was also observed to decrease in the subcutaneous adipose tissue of mice fed with a high fat diet [16]. For the IL–33 receptor, the expression of the suppression of tumorigenicity2 (ST2) gene was also increased in the adipose tissue of obese mice and humans [14]. Moreover, in omental and subcutaneous adipose tissue from severely obese humans, IL–33 expression was increased approximately threefold compared to lean counterparts, which was also confirmed at the protein level in obese humans and mice [14].

On the other hand, IL–37 is a novel cytokine which has modulatory effects on immune responses and has anti-inflammatory functions through three mechanisms, i.e., by reducing the expression of transcriptional cytokines, by inhibiting the activation of kinase signaling, and by decreasing the production of pro-inflammatory cytokines [17,18]. Several human tissues and organs that express IL–37 include the heart, skin, gut, kidney, thymus, lymph node, lung, bone marrow, uterus, testis, and placenta [19]. Under physiological conditions, IL–37 is expressed at low levels and can be upregulated in response to inflammatory stimuli. For instance, in Toll-like receptor (TLR) activation and lipopolysaccharide (LPS) treatment, IL–37 is subsequently released by macrophages [20,21]. In addition, IL–37 acts to restore cell metabolic homeostasis during inflammation and reverse chronic inflammation of metabolic stress [22,23]. Interestingly, there is no or little data about the relationship between IL–33 and IL–37 in individuals with MetS. 

Since IL–37 is considered to be an endogenous anti-inflammatory protein involved in various physiological and pathological processes, its role in the inflammatory state associated with MetS merits investigation. Hence, the present cross-sectional study investigated the differences in serum levels of IL–33 and IL–37 in adults with or without MetS and their relationship with MetS components. Additionally, we investigated if sexual dimorphism exists in the levels of circulating ILs–33 and–37 of adults with and without MetS.

## 2. Results

The clinical characteristics of all participants are shown in Table 1, stratified by sex, while characteristics according to MetS status was presented in Table S1. The National Cholesterol Education Program Adult Treatment Panel III (NCEP ATP III) criteria [24] was used to screen participants for MetS. The prevalence of MetS was higher among females (48.9%) than males (31.1%), *p <* 0.001. All MetS parameters were significantly higher in females than in males, except for hypertriglyceridemia which was more prevalent among males. Furthermore, males had higher IL-33 levels than females [males; 3.7 pg/mL (3.0–4.1) vs. females; 3.2 pg/mL (1.4–3.8), *p <* 0.001]. IL–37 was also higher in females than in males, but the significance was modest [females; 3.01 (2.2–7.0) vs. males; 2.9 (2.1–5.6), *p* = 0.06]. 

**Figure 1:**
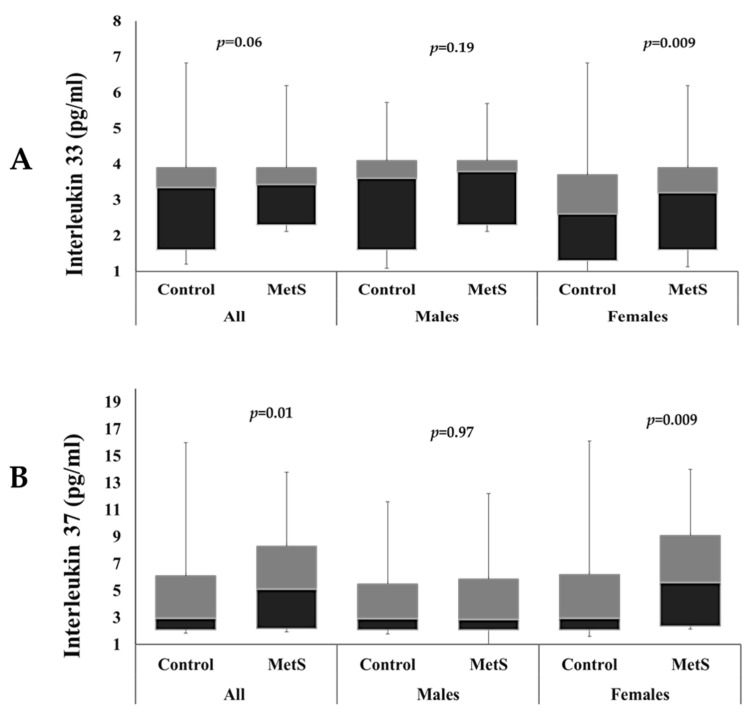
Comparison of circulating IL–33 levels (**A**) and IL–37 levels (**B**) among participants with and without MetS.

Figure 1 shows the differences in IL–33 and IL–37 levels among participants with and without MetS in both sexes. IL–33 levels were significantly higher in females with MetS than female controls (*p* = 0.009). Serum levels of IL–37 were also significantly higher in MetS group than controls overall (*p* = 0.01), and in females with MetS than in control females (*p* = 0.009). Both ILs were not significantly different between males with and without MetS.

In Table 2, serum IL–33 and IL–37 levels are classified into tertiles, with tertile one being the lowest and tertile three the highest. Logistic regression analysis was performed with adjustment for covariates. Models 1, 2, 3, and 4 were used as follows; model 1 was univariate, model 2 was adjusted for age, model 3 was adjusted for age and BMI, and model 4 was adjusted for age, BMI, and sex. The results showed that higher tertiles of IL–33 serum levels were associated with higher odds of high blood pressure, low HDL-c, and hypertriglyceridemia (*p*-values < 0.01), and this remained significant in all models. For IL–37, the highest tertile was associated only with hyperglycemia (*p* < 0.01) and this was statistically significant in all models. While the mid tertile of IL–37 was associated with higher odds of hyperglycemia (*p* < 0.01) and low HDL-c (*p* < 0.05), which was statistically significant in all models. As for full MetS, the results showed that higher tertiles of IL–33 levels were associated with higher odds of having full MetS (*p* < 0.01) in all models. IL–37 showed no significant associations with full MetS after adjustments.

Table 3 shows the bivariate associations of IL–33 and IL–37 with measured parameters. In the MetS group, IL–33 had statistically significant inverse correlations with age (−0.25, *p* < 0.01), glucose (−0.18, *p* < 0.05), and HDL (−0.28, *p* < 0.01), and significant positive correlation with triglycerides (0.17, *p* < 0.05). IL–37 only had significant inverse correlation with triglycerides in the MetS group (−0.23, *p* < 0.05). In the control group, the results showed that IL–33 had statistically significant inverse correlations with age (−0.15, *p* < 0.05), TC (–0.14, *p* < 0.01), and HDL (−0.51, *p* < 0.01), and a significant positive correlation with SBP (0.24, *p* < 0.01) and triglycerides (0.14, *p* < 0.05). While IL–37 had significant positive correlation with triglycerides (−0.16, *p* < 0.05) in the control group.

Lastly, stepwise logistic regression analysis using IL-33 and IL–37 (Table 4) as the dependent variables showed that age, SBP, glucose, and HDL-c explain 24% of variations in circulating IL–33 levels among all participants. In males, HDL-c explains 29% of variations in circulating IL–33 levels (*p* < 0.001), while results for the females showed that age (*p* = 0.007) and HDL-c (*p* < 0.0001) explain 25% of variations in circulating IL–33 levels. For IL–37, the stepwise logistic regression analysis showed no significant predictors.

## 3. Discussion

The present study investigated the factors associated with serum levels of IL–33 and IL–37 in Saudi Arabian adults with and without MetS. Higher levels of serum IL–33 and IL–37 were seen in individuals with MetS compared to controls. Moreover, in individuals with high levels of IL–33 and IL–37, we observed significantly more pronounced characteristics of MetS simultaneously. When an analysis was performed on the association of IL–33 with different MetS components, we found that high levels of circulating IL–33 is a risk factor for MetS components; namely, hypertension, low HDL-c, and hypertriglyceridemia.

The primary event in MetS development is chronic positive calorie intake with an increase in adipose tissue mass [25]. IL–33, a newly identified cytokine, has drawn much attention for its ability to regulate multiple immune responses and its involvement in the pathogenesis of several diseases [26,27]. Evidence for the correlation between circulating levels of IL–33 and IL–37 in MetS and its associated metabolic disorders in human adults is scarce. Our results indicated that serum IL–33 levels had a significant inverse correlation with HDL-c among participants with MetS and that higher tertiles of IL–33 were associated with higher odds of having low levels of HDL-c. This is in line with Tang et al., who indicated an inverse correlation between IL–33 and HDL-c in overweight/obese subjects [28]. Our results therefore suggest that IL–33 is closely related to risk factors for CVD, e.g., blood lipid level and blood pressure.

Furthermore, in the present results, IL–33 levels had a significant association with triglycerides overall. Recently, Gorzelak et al. evaluated the effects of a single fat-rich meal on inflammatory status and barrier functions in human umbilical vascular endothelial cells (HUVECs), and demonstrated an almost 2.5-fold increase in IL–33 mRNA expression secondary to postprandial hypertriglyceridemia in HUVECs [29]. Consequently, the ability to control postprandial lipemia is of significant clinical interest, not only amongst individuals at risk for CVD but also in healthy individuals [29].

Here, we found that IL–33 negatively affected the circulating levels of glucose in adults with MetS. The first step of glucose metabolism is glucose uptake through glucose transporters (GLUTs) [30,31]. Previously, it has been shown that glycolysis and GLUT1 expression were upregulated in the IL–33/ST2 pathway in non-small cell lung cancer patients compared to control tissues [32]. Recently, studies found that the treatment of mast cells with IL–33 promoted glycolysis and had a significant role in the recruitment of neutrophils in a glucose-dependent manner [33,34]. Additionally, the phenomenon of the Warburg effect, which is characterized by high rates of glucose uptake, was also commonly observed in fast growing cancer cells [35,36]. Recently, Liang et al. demonstrated an increase in glucose uptake in CD8 effector T cells after treatment with IL–33 [37]. The authors found that IL–33 treatment increased the expression and transcription level of glucose transporter GLUT1 in several glycolytic enzymes. However, Pereira et al. recently reported that the treatment of subcutaneous adipose tissues with IL–33 ex-vivo decreased glucose uptake in isolated adipocytes, where analyses of IL–33 expression showed positive associations between IL–33, insulin resistance, obesity, and T2DM [38]. This is in line with previous research which showed that the circulating levels of IL–33 and its expression in adipose tissue were increased in individuals with metabolic disorders and obesity [39,40]. This contradiction suggests a more complex relationship between IL–33 and the progression of MetS. The cause of this inconsistency is unknown, but levels of circulating and tissue IL–33 are not necessarily coherent as numerous lining and structural cells, including endothelial cells, epithelial cells, and fibroblasts, in control human tissues including the gastrointestinal tract, blood vessels, lungs, and liver constitutively produce IL–33 [41]. Whereas, IL–33’s distribution pattern or physiological function is closely related to its production site and secretion mode [42,43]. Therefore, IL–33’s pathophysiological properties could rely on its producing cell and temporal expression [42]. Consequently, the relationship between the serum levels of IL–33 and its tissue expression in different depots could be more complicated. 

We found significant differences in serum IL–33 levels between males and females in our study population. Previously, it was demonstrated that IL–33 production was induced by estradiol-dependent signals using Erα [44], while Momen et al. reported no significant disparities in IL–33 levels among males and females [45]. Additionally, Zhao et al., in a murine model, demonstrated sex differences in response to IL–33 treatment in IL–33-induced airway inflammation, and the authors found that type 2 inflammation induced by treatment with ovalbumin + IL–33 was more severe in female mice compared to males [46]. Moreover, Peng et al. demonstrated a sex differences in IL–33 mRNA levels within the heart tissues of mice, with increased levels of IL–33 mRNA in female mice compared to male mice [47].

Our results showed an inverse correlation between IL-37 and triglycerides in the MetS group. A previous transgenic mouse model study demonstrated the regulatory role of IL–37 in lipid homeostasis [48], in transgenic mice for human IL–37, after 16 weeks of high fat diet. These mice showed reduced levels of plasma triglycerides, cholesterol, and fatty acids, when compared with wild mice. In the same study, in vitro adipocytes treatment with recombinant IL–37 decreased adipogenesis and activated signaling of AMP-activated protein kinase. Moreover, studies have reported that treatment with IL–37 recombinant reduces the formation of foam cells and the accumulation of lipids which turn into atherosclerotic plaque [49,50,51].

Our data showed elevated levels of circulating IL–37 in individuals with MetS along with elevated levels of IL–33, suggesting that IL–37 could play a role in the modulation of the inflammatory states associated with MetS. IL–37 functions as an anti-inflammatory cytokine following inflammatory stimuli via the IL1-R5/IL-1R8 receptor complex [52,53]. Extracellular IL–37 acts as an anti-inflammatory in different ways; (1) signaling of mitogen-activated protein kinase (MAPK) [52,53,54], (2) activation of NF-B [54], (3) suppressing cytokine production [55], (4), metabolic signaling by molecular target of rapamycin (mTOR) [56], and (5) mediators associated with ERK and p38 [55]. At the same time, a central metabolic regulator and anti-inflammatory mediator such as AMPK is activated [57]. Nevertheless, with increasing IL–37 protein concentrations the anti-inflammatory effects fade [54]; this could be due to homodimers formation [58].

The authors acknowledge some limitations. The sample size in the present study was relatively small and multiple stratifications may have increased the likelihood of type 2 errors. In addition, causality cannot be assessed given the limitations of the present study’s design. Follow-up studies are needed, in which the levels of IL–33 and IL–37 at multiple time points are required. Nevertheless, the present study sheds new light on circulating IL–33 and IL–37 levels in individuals with or without MetS, as well as the relationship between the investigated ILs and MetS components.

## 4. Materials and Methods

### 4.1. Participants

A total of 417 Saudi adults aged 30–50 years [266 (64%) females] were randomly selected from teachers of 60 preparatory and high schools in Riyadh City, Saudi Arabia. Inclusion criterion was consenting males and females. Exclusion criteria were those participants with malignancy, lung, or cardiac diseases, etc., which required immediate medical attention. All participants completed a questionnaire on demographic information, general health status, and past medical history. Written informed consent was obtained from all participants before their inclusion in this study. 

### 4.2. MetS Criteria 

NCEP ATP III criteria [24] were used to screen participants for MetS. A person with at least three of the following five risk factors was considered a MetS patient:

(1) Waist circumference (Central obesity) of >101.6 cm in males and >88.9 cm in females.
(2) Fasting glucose (Hyperglycemia) > 5.6 mmol/L.
(3) Low high-density lipoprotein cholesterol (HDL-c); <1.03 mmol/L for males and <1.30 mmol/L for females.
(4) Fasting triglycerides (Hypertriglyceridemia) >1.7 mmol/L.
(5) Hypertension; diastolic blood pressure >85 mmHg; and/or systolic blood pressure >130 mmHg.

### 4.3. Anthropometrics and Biochemical Analyses

Participants were instructed to come to a fasting state; anthropometric measures and blood samples were collected from all participants by trained nurses. Height (cm), weight (kg), systolic and diastolic blood pressures (SBP and DBP, respectively), waist and hip circumferences (cm) were measured using routine methods by trained nurses [59]. Body mass index (BMI; kg/m^2^) and waist–hip ratio (WHR) were calculated. Lipid profile and blood glucose levels were measured using an automated biochemical analyzer (Konelab 20 Thermo-Fischer, Espoo, Finland) using commercially available kits (catalogue nos. 981379, 981812, 981823 and 981301, respectively) [60]. IL–33 serum levels were measured using available commercial ELISA kit (BioVendor, R&D systems, Brno, Czech Republic) Cat No. RAF064R [61,62]. According to the manufacturer, intra-assay and inter-assay % CV were less than 4.7% and less than 6.9%, respectively. IL–37 serum levels were measured using Flex MAP 3D System (Luminex Corporation, Austin, TX, USA) using human cytokines Magnetics Bead Panels Cat No. HCYP4MAG-64K Human Cytokine/Chemokine Magnetic Bead Panel IV. Intra-assay and inter-assay % CV were <10 and <15, respectively, according to the manufacturer. 

### 4.4. Statistical Analysis

Data were analyzed using SPSS (version 22 Chicago, IL, USA). Continuous data were presented as mean ± standard deviation (SD) for Gaussian variables, and non-Gaussian variables were presented in median (25st and 75th) percentiles. Categorical data were presented as frequencies and percentages N (%) (i.e., central obesity, hypertension, low HDL-c, hypertriglyceridemia, hyperglycemia). All continuous variables were checked for normality using Kolmogorov–Smirnov test. Non-Gaussian variables were log-transformed prior to parametric analyses. Mann–Whitney U test was performed to find median differences in IL-33 and IL-37. Correlation analyses were performed to determine bivariate associations of IL–33 and IL–37 with other parameters and presented as coefficients (R). Multinomial logistic regression was used to check for factors associated with IL–33 and IL–37 levels classified into tertiles and the odds of having different MetS components were calculated and adjusted for covariates [model 1 univariate, model 2 adjusted with age, model 3 adjusted with age and BMI, and model 4 adjusted with age, BMI, and gender]. Stepwise regression analysis was performed to check associations between IL–33 and IL-37 as dependent variables and age, BMI, waist, hip, SBP, DBP, glucose, total cholesterol, HDL-c, and triglycerides as independent predictors. A *p*-value < 0.05 was considered statistically significant. 

## 5. Conclusions

The present study indicated that serum levels of IL-33 and IL-37 were higher in MetS individuals than controls, were sexually dimorphic, and were associated with individual MetS components. IL-33 could be a novel therapeutic target to prevent MetS progression, but more studies are needed to determine IL-37’s role in MetS development. Follow-up studies can confirm present findings.

## Figures and Tables

**Table 1:** Clinical characteristics of our study participants.

Parameters	All (N = 417)	Males (N = 151)	Females (N = 266)	*p*-Value
MetS N(%)	177 (42.4)	47 (31.1)	130 (48.9)	<0.001
Central obesity N(%)	171 (41.0)	38 (25.2)	133 (50.0)	<0.001
Hypertension N(%)	115 (27.6)	38 (25.2)	77 (28.9)	0.24
Low HDL–c N(%)	243 (58.3)	76 (50.3)	167 (62.8)	0.009
Hypertriglyceridemia N(%)	151 (36.2)	63 (41.7)	88 (33.1)	0.05
Hyperglycemia N(%)	219 (52.5)	64 (42.4)	155 (58.3)	0.001
Age (years)	41.3 ± 9.0	40.0 ± 9.7	42.1 ± 8.6	0.02
BMI (kg/m^2^)	30.7 ± 6.3	29.1 ± 5.4	31.5 ± 6.7	<0.001
Waist (cm)	91.4 ± 17.2	92.7 ± 19.8	90.6 ± 15.6	0.26
Hip (cm)	105.1 ± 18.0	101.1 ± 20.0	107.2 ± 16.5	0.002
WHR	0.87 ± 0.10	0.92 ± 0.10	0.85 ± 0.10	<0.001
Systolic BP (mmHg)	122.3 ± 13.9	122.5 ± 12.8	122.1 ± 14.6	0.76
Diastolic BP (mmHg)	76.3 ± 10.1	75.9 ± 9.5	76.5 ± 10.4	0.52
Glucose (mmol/L)	6.2 ± 2.1	5.9 ± 1.9	6.4 ± 2.2	0.02
Total Cholesterol (mmol/L)	5.20 ± 1.1	5.21 ± 1.03	5.20 ± 1.1	0.90
HDL-cholesterol (mmol/L)	1.19 ± 0.4	1.08 ± 0.31	1.25 ± 0.4	<0.001
Triglycerides (mmol/L)	1.4 (1.0–2.0)	1.5 (1.1–2.1)	1.4 (1.0–1.9)	0.04
IL–33 (pg/mL)	3.4 (1.4–3.9)	3.7 (3.0–4.1)	3.15 (1.4–3.8)	<0.001
IL–37 (pg/mL)	2.9 (2.2–6.2)	2.9 (2.1–5.6)	3.01 (2.2–7.0)	0.06

Note: Data presented as mean ± SD. *p*-value significant at 0.05 level.

**Table 2:** Logistic regression analysis for MetS and its components in tertiles of circulating IL–33 and IL–37.

MetS Component	IL–33				IL–37		
	Odd Ratio (95%CI)				Odd Ratio (95%CI)		
	Model	Tertile 1 (<2.37)	Tertile 2 (2.37–3.78)	Tertile 3 (>3.78)	Tertile 1 (<2.68)	Tertile 2 (2.68–5.67)	Tertile 3 (>5.67)
Central Obesity	1	1	1.12 (0.67–1.86)	1.04 (0.62–1.73)	1	1.11 (0.6–1.98)	1.45 (0.8–2.6)
	2		1.24 (0.73–2.09)	1.13 (0.67–1.92)		1.10 (0.61–1.97)	1.39 (0.78–2.5)
	3		0.99 (0.53–1.86)	1.11 (0.6–2.04)		1.18 (0.6–2.31)	1.17 (0.61–2.26)
	4		1.10 (0.58–2.10)	1.42 (0.75–2.70)		1.20 (0.6–2.41)	1.10 (0.56–2.13)
Hypertension	1	1	1.67 (0.93–3.0)	1.71 (0.95–3.1)	1	0.78 (0.40–1.50)	1.10 (0.58–2.1)
	2		1.88 (1.03–3.45) *	1.91 (1.1–3.5) **		0.76 (0.39–1.48)	0.96 (0.50–1.85)
	3		1.74 (0.94–3.25)	1.97 (1.1–3.6) *		0.71 (0.36–1.43)	0.80 (0.41–1.57)
	4		1.82 (0.97–3.39)	2.15 (1.1–4.1) *		0.71 (0.35–1.43)	0.77 (0.39–1.53)
Hyperglycemia	1	1	1.63 (0.99–2.7)	1.22 (0.74–2.0)	1	0.38 (0.21–0.69) **	2.59 (1.4–4.71) **
	2		1.90 (1.12–3.22) *	1.38 (0.82–2.32)		0.36 (0.20–0.67) **	2.44 (1.3–4.48) **
	3		1.64 (0.94–2.88)	1.16 (0.67–2.02)		0.35 (0.18–0.67) **	2.51 (1.32–4.8) **
	4		1.74 (0.98–3.10)	1.29 (0.73–2.28)		0.34 (0.18–0.67) **	2.57 (1.3–4.92) **
Low HDL-cholesterol	1	1	7.94 (4.5–14.1) **	3.79 (2.2–6.4) **	1	0.49 (0.28–0.87) *	1.02 (0.6–1.8)
	2		8.81 (4.9–15.9) **	4.11 (2.4–7.1) **		0.48 (0.27–0.87) *	0.97 (0.54–1.72)
	3		8.59 (4.7–15.8) **	4.1 (2.3–7.1) **		0.48 (0.26–0.88) *	0.88 (0.48–1.60)
	4		10.5 (5.5–19.9) **	5.34 (2.9–9.7) **		0.47 (0.25–0.88) *	0.82 (0.45–1.52)
Hypertriglyceridemia	1	1	2.41 (1.4–4.2) **	3.13 (1.8–5.5) **	1	0.76 (0.41–1.41)	1.35 (0.75–2.42)
	2		2.64 (1.5–4.7) **	3.42 (1.9–6.1) **		0.75 (0.40–1.39)	1.24 (0.68–2.26)
	3		2.51 (1.4–4.5) **	3.22 (1.8–5.7) **		0.78 (0.41–1.50)	1.19 (0.64–2.21)
	4		2.45 (1.4–4.4) **	3.05 (1.7–5.5) **		0.78 (0.40–1.49)	1.26 (0.67–2.37)
Full MetS	1	1	1.95 (1.2–3.3) *	1.82 (1.1–3.1) *	1	0.69 (0.38–1.27)	1.86 (1.1–3.32) *
	2		2.42 (1.4–4.2) **	2.22 (1.3–3.9) **		0.67 (0.36–1.25)	1.69 (0.94–3.10)
	3		2.34 (1.3–4.3) **	2.26 (1.2–4.1) **		0.65 (0.33–1.29)	1.45 (0.76–2.73)
	4		2.58 (1.4–4.8) **	2.79 (1.5–5.2) **		0.65 (0.33–1.29)	1.39 (0.73–2.64)

Note: Data from the logistic regression analysis were presented as odds ratio, 95% confidence interval, associated *p*-value. Odds ratio for higher IL–33 and IL–37 tertiles (2 and 3) was calculated by taking tertile 1 as reference (represented by value 1). Models 1, 2, 3, and 4 are univariate, + adjusted with age, and + adjusted with BMI, and + adjusted with gender, respectively. * and ** represent *p*-value significance, and *p* < 0.01 and 0.05 were taken as significant.

**Table 3:** Correlation analysis of IL–33 and IL–37 with measured parameters.

Parameters	Control		MetS	
	IL–33	IL–37	IL–33	IL–37
IL–33 (pg/mL)	--	0.07	--	−0.14
IL–37 (pg/mL)	0.07	--	−0.14	--
Age (years)	−0.15 *	−0.07	−0.25 **	0.06
BMI (kg/m^2^)	0.01	0.11	−0.07	0.04
Waist (cm)	0.06	0.01	−0.11	0.00
WHR	−0.04	−0.06	−0.02	0.06
Systolic BP (mmHg)	0.24 **	−0.01	0.00	0.05
Diastolic BP (mmHg)	0.12	−0.05	0.04	−0.04
Glucose (mmol/L)	0.12	0.09	−0.18 *	0.01
Total Cholesterol (mmol/L)	−0.14 *	0.11	0.02	−0.05
HDL-c (mmol/L)	−0.51 **	0.06	−0.28 **	0.09
Triglycerides	0.14 *	0.16 *	0.17 *	−0.23 *

Note: Data presented as coefficient (R). * and ** represents *p*-value significance at 0.05 and 0.01 levels, respectively.

**Table 4:** Stepwise regression analysis for IL-37 and IL–33.

IL–33			
	All	Males	Females
Adjusted R^2^	0.24	0.29	0.25
Unstandardized Co-efficient	β ± S.E, *p*-value	β ± S.E, *p*-value	β ± S.E, *p*-value
Age	−0.024 ± 0.01	---	−0.03 ± 0.01, *p* = 0.007
SBP	0.02 ± 0.007, *p* = 0.008	---	---
Glucose	−0.10 ± 0.05, *p* = 0.039	---	---
HDL–c	−1.73 ± 0.24, *p* < 0.001	−2.97 ± 0.65, *p* < 0.001	−1.65 ± 0.24, *p* < 0.001

Note: Independent predictors: age, BMI, waist, hip, systolic and diastolic BP, glucose, cholesterol, HDL-c, triglycerides, and IL–33.

## Data Availability

The data presented in this study are available on request from the corresponding author. The data are not publicly available due to privacy protection.

## Supplementary Materials

The supporting information can be downloaded at: https://www.mdpi.com/article/10.3390/ijms25020699/s1.
