# Supplementary material for: Circulating Interleukins-33 and -37 and Their Associations with Metabolic Syndrome in Arab Adults
Source: Int J Mol Sci. 2024 Jan 5;25(2):699. doi: 10.3390/ijms25020699 (PMC10815042; doi:10.3390/ijms25020699)
Supplement: Supplementary file 1 [file ijms-25-00699-s001.zip › ijms-2785614-supplementary.pdf]

Supplementary table S1: Clinical Characteristic of the subjects.

Parameters	All		Age and BMI adjusted P-value	Males		Age and BMI adjusted P-value	Females		Age and BMI adjusted P-value
	Control	MetS		Control	MetS		Control	MetS	
N	240	177		104	47		136	130	
Age	39.4±9.2	44.0±8.1		38.6±9.4	43.1±9.7		39.9±8.9	44.3±7.5	
BMI (kg/m^2)	28.7±5.5	33.2±6.5		28.6±5.1	30.3±5.9	0.072	28.9±5.8	34.2±6.4	
Waist (cm)	84.7±16.2	99.6±14.8	<0.001	89.1±17.5	100.3±22.3	0.025	81.4±14.4	99.3±11.2	<0.001
WHR	0.86±0.10	0.89±0.10	0.001	0.90±0.10	0.95±0.10	0.021	0.82±0.09	0.87±0.08	<0.001
Systolic BP	117.7±11.1	128.3±15.0	<0.001	119.2±9.6	129.9±15.6	<0.001	116.6±11.9	127.7±14.8	<0.001
Diastolic BP	73.8±9.2	79.5±10.3	<0.001	74.3±8.6	79.3±10.5	0.015	73.5±9.7	79.5±10.3	<0.001
Glucose (mmol/l)	5.6±1.9	7.1±2.1	<0.001	5.6±2.0	6.5±1.4	0.035	5.5±1.9	7.3±2.3	<0.001
HbA1C (%)	5.5±1.2	6.2±1.2	<0.001	5.5±0.9	5.8±1.0	0.168	5.5±1.4	6.3±1.3	<0.001
Insulin (uU/ml)	13.3±6.8	17.4±5.2	<0.001	13.2±8.5	19.1±4.6	0.020	13.4±5.6	16.9±5.3	0.026
Chol (mmol/l)	5.2±1.0	5.1±1.1	0.314	5.3±1.0	5.1±1.1	0.282	5.2±1.1	5.2±1.1	0.582
HDL (mmol/l)	1.31±0.4	1.02±0.3	<0.001	1.14±0.3	0.93±0.2	<0.001	1.45±0.4	1.05±0.3	<0.001
Triglycerides	1.24 (0.9-1.6)	1.86 (1.3-2.4)	<0.001	1.33 (1.0-1.8)	2.0 (1.7-2.5)	<0.001	1.14 (0.9-1.5)	1.8 (1.3-2.3)	<0.001
IL-33 (pg/ml)	3.34 (1-3.9)	3.42 (2.3-3.9)	0.057	3.6 (1.6-4.1)	3.79 (3.5-4.1)	0.187	2.6 (0.9-3.7)	3.2 (1.6-3.9)	0.009
IL37 (pg/ml)	2.92 (2.1-6.1)	5.11 (2.2-8.3)	0.012	2.9 (2.1-5.5)	2.83 (2.1-5.87)	0.973	2.92 (2.1-6.2)	5.6 (2.4-9.1)	0.009

Note: Data Presented mean ± SD. P-value significant at 0.01 and 0.05 level.
